# Rethinking ADHD as a neurointestinal syndrome: a gut–brain–parasite hypothesis

**DOI:** 10.3389/fnins.2025.1694628

**Published:** 2026-01-14

**Authors:** Alexis Demas

**Affiliations:** 1 Neurology Unit, Department of Neurology, Le Havre Hospital, Le Havre, France; 2 ARGOS (Art, Research and Gestures Observed Scientifically) Neurosciences and Arts, Paris, France

**Keywords:** ADHD, gut, helminth, gut–brain axis, microbiota

## Abstract

Neurodevelopmental conditions such as Attention-Deficit/Hyperactivity Disorder (ADHD) are usually framed as brain-based disorders driven by genetics and neurotransmitter imbalance. At the same time, converging evidence implicates the gut–brain axis and intestinal immunity in shaping cognition and behavior. In this Hypothesis and Theory article, I propose that a subset of ADHD and related neurodivergent profiles can be usefully conceptualized as neurointestinal syndromes, emerging from co-evolutionary interactions between the gut microbiota, intestinal parasites, and host immunity. Drawing on data from ADHD, autism spectrum conditions, and migraine, I synthesize evidence for altered microbiota, increased intestinal permeability, and low-grade inflammation in neurodivergent individuals, and discuss how these changes may bias tryptophan metabolism, vagal signaling, and large-scale brain networks. I then explore a speculative evolutionary scenario in which recurrent helminth exposure, historically ubiquitous, acted as a long-term ecological force shaping gut architecture, immunoregulation, and stress responsivity. Chronic parasitic pressure, combined with microbial metabolites and epigenetic imprinting, may have contributed to the emergence of attentional profiles characterized by hypervigilance, novelty seeking, and rapid switching—traits that could have been advantageous in ancestral, pathogen-rich environments but are often maladaptive in modern settings. This framework does not romanticize ADHD nor deny its frequent clinical burden. Rather, it reframes some ADHD phenotypes as possible mismatch syndromes involving the gut–brain axis, generated when an evolutionarily tuned intestinal and immune architecture is placed in sanitized, post-industrial ecologies. Clinically, this perspective supports continued use of established CNS-targeted treatments while motivating complementary research into microbial, barrier, and vagal interventions as potential adjuncts for carefully defined ADHD subgroups.

## 1 Introduction: from brain-centric to gut-centric neurodivergence

For decades, conditions such as Attention Deficit Hyperactivity Disorder (ADHD), autism spectrum disorder (ASD), and migraine have been predominantly conceptualized as brain-centric pathologies—disorders of cortical structure, neurotransmitter imbalances, or disrupted neurodevelopmental trajectories. However, a growing body of evidence reveals consistent, although heterogeneous, abnormalities in the gastrointestinal tract of many neurodivergent individuals, including alterations in gut microbial composition, increased intestinal permeability, dysregulated mucosal immunity, and low-grade systemic inflammation.

This convergence has led researchers to propose a “gut–brain axis” model, in which bidirectional signaling between the enteric and central nervous systems plays a central role in shaping behavior and cognition (Cryan and Dinan, 2012; Cryan and O'Mahony, 2011). Recent high-quality reviews on the microbiome–gut–brain axis and neurodevelopment emphasize that this communication is multimodal (neural, immune, endocrine, metabolic), active throughout development, and capable of exerting long-term effects on cognitive and emotional trajectories (Vuong and Hsiao, 2017; Stiemsma and Michels, 2018; Bastiaanssen and Cryan, 2021; Ramadan et al., 2025).

In children with ADHD, several studies report lower microbial alpha-diversity and enrichment of specific genera such as *Bacteroides* and *Bifidobacterium*, potentially influencing dopamine pathways via microbial metabolites like phenylalanine and short-chain fatty acids (SCFAs) (Aarts et al., 2017; Prehn-Kristensen et al., 2018; Alverina et al., 2021). Similarly, children with autism often display increased gastrointestinal symptoms, altered tryptophan metabolism, and microbial profiles associated with immune dysregulation (Kang et al., 2013; Sharon et al., 2019). A recent review of the gut–brain axis in autism further supports the view that microbial dysbiosis and barrier changes are relevant for a subset of ASD and ADHD cases (Hetta et al., 2025).

These gut alterations are not easily dismissed as epiphenomena. Zonulin-mediated disruption of tight junctions, elevated intestinal cytokines, and microbiota-induced breakdown of immune tolerance have been documented across multiple neurodivergent cohorts (Fasano, 2012; Magistris et al., 2010). Notably, similar abnormalities are found in migraine—a condition traditionally classified as a primary neurological disorder. Migraineurs often display gut dysbiosis, increased gut permeability, and comorbid inflammatory bowel conditions, suggesting a shared enteric substrate with ADHD and ASD (Martami et al., 2018; Arzani et al., 2020).

These observations motivate a shift in how we understand at least some forms of neurodivergence: not only as defects originating in the brain, but as systemic phenotypes rooted in the ecology of the gut. This Perspective takes the idea a step further. I hypothesize that certain neurodivergent traits may have co-evolved with parasitic and microbial pressures in ancestral environments, and that the gut, far from being a passive victim of brain dysfunction, may be an original site of divergence. Helminths, protozoa, and bacteria may not merely complicate neurodivergent presentations; they may have helped shape them (Rook, 2010; Parker and Ollerton, 2013).

To guide the reader, the manuscript proceeds as follows. Section II considers the gut as an “architect” of neurodevelopment through microbial inheritance and early-life programming. Sections III and IV discuss helminths as evolutionary sculptors and hidden modulators of the gut–brain–immune axis. Section V addresses epigenetic and transgenerational aspects of intestinal “memory”. Section VI frames the hypothesis in evolutionary terms, with explicit attention to its limitations. Section VII applies this framework to ADHD as a candidate neurointestinal profile, and Section VIII concludes by outlining testable predictions and clinical implications.

This hypothesis reframes aspects of neurodivergence as not only neurodevelopmental conditions, but whole-body ecological expressions of co-evolutionary trade-offs. The gut becomes not a secondary actor in brain disorders, but a historical stage upon which some of the foundational dramas of cognition, immunity, and vulnerability were enacted.

## 2 The gut as architect: microbial inheritance and cortical divergence

Infancy is not merely a phase of cortical wiring; it is also the primal moment of microbial inscription. From birth, neonates inherit a maternal microbiota that profoundly shapes the immune system, modulates neuromediator synthesis, and calibrates stress responsiveness (Vuong and Hsiao, 2017; Gensollen et al., 2016; Vuong and Hsiao, 2017). This microbial transmission influences not only local gut physiology but also microglial maturation and synaptic pruning in the developing brain, establishing an early bidirectional dialogue between gut and cortex (Cryan and O'Mahony, 2011; Erny et al., 2015).

In individuals with ADHD, this microbial signature appears to diverge in many, though not all, studies. Several reports describe reduced microbial alpha-diversity and alterations in specific taxa, such as increased *Clostridium* and *Bacteroides* species, alongside a depletion of SCFA producers (Aarts et al., 2017; Prehn-Kristensen et al., 2018; Alverina et al., 2021). Findings remain heterogeneous and effect sizes are often modest, yet they converge on the idea that, in a subset of patients, microbial communities differ in ways that *could* influence neurodevelopment.

SCFAs such as butyrate and propionate play critical roles in maintaining intestinal barrier integrity, modulating vagal afferents, and regulating the synthesis of key neurotransmitters including GABA and serotonin (Dalile et al., 2019; Yano et al., 2015; Stilling et al., 2016). Altered SCFA profiles, combined with reduced barrier function, may promote low-grade inflammation and modify sensory and emotional processing.

One particularly important axis lies in tryptophan metabolism. Altered gut flora in ADHD and ASD patients can destabilize the kynurenine pathway, leading to changes in neuroactive metabolites that may modulate arousal, attention, and mood, albeit in complex and context-dependent ways (Joanna et al., 2017). Rather than implying a direct and uniform causal chain, current data suggest that gut-derived metabolites are one set of modulators among many of cortico-limbic connectivity patterns, especially in frontal and striatal regions implicated in executive function.

Moreover, impaired vagal signaling due to gut dysbiosis and inflammation may influence the default mode network (DMN) and large-scale brain networks critical for attention switching and cognitive flexibility (Bonaz et al., 2018; Menon, 2011). In this framework, the gut is not a passive reactor but a co-author in the formation of attentional circuits and emotional tone.

Importantly, this architecture is plastic and shaped by bidirectional influences. Diet, stress, sleep, physical activity, and medications (including antibiotics and psychostimulants) continuously remodel the microbiota, which in turn modulates immune and neural signaling (Dalile et al., 2019; Foster et al., 2017). Chronic psychosocial stress and irregular sleep in children with ADHD may therefore both reflect and reinforce gut–brain dysregulation, rather than being purely downstream consequences.

This early microbiota signature may even carry an epigenetic imprint. Maternal stress, infection, or dietary patterns influence not only microbial inheritance but also gene expression in the neonate via epigenetic pathways modulated by microbial metabolites (Stiemsma and Michels, 2018; Ramadan et al., 2025; Jaenisch and Bird, 2003). Recent reviews on the microbiome–gut–brain axis underscore how microbial composition and host epigenetic mechanisms jointly shape brain development and mental health across the lifespan (Bastiaanssen and Cryan, 2021; Guangxu et al., 2018). In this light, the gut is the first teacher—writing, in microbial ink, the prolog of a neurocognitive trajectory.

## 3 Parasites as evolutionary sculptors: helminthic exposure and neurodivergent resilience

While bacteria and metabolites shape neurodevelopment on a microscopic scale, an even older evolutionary player, the intestinal parasite, may have guided the emergence of certain neurodivergent traits on a macroscopic timescale. Helminths, particularly soil-transmitted nematodes, have coexisted with humans for hundreds of millennia. Far from being mere pathogens, these organisms imposed a powerful selective pressure on the immune system, endocrine signaling, and possibly behavior (Rook, 2010; Parker and Ollerton, 2013; Reynolds et al., 2015; Bilbo et al., 2018).

In ancestral environments, recurrent helminth exposure shaped gut ecology in ways that enhanced tolerance and immunoregulation. Helminths secrete immunomodulatory molecules, skewing host immunity toward Th2 and regulatory T-cell responses, dampening inflammation while often promoting gut barrier integrity and microbial diversity (Menon, 2011; Maizels, 2020; Maizels and Yazdanbakhsh, 2003; Maizels and McSorley, 2016; Rescigno, 2017). Their presence appears to have stabilized microbial ecosystems, limiting overgrowth of pro-inflammatory taxa and supporting the growth of SCFA-producing commensals (Reynolds et al., 2015; Bilbo et al., 2018).

In neurodevelopmental terms, this cohabitation may have buffered early-life inflammation, supported serotonergic homeostasis, and preserved gut–brain vagal tone. Such conditions could, in principle, contribute to cognitive profiles more adapted to environmental unpredictability, though direct evidence in humans remains limited. It is therefore important to present this as a speculative scenario: the presence of helminths *could* have constrained maladaptive neuroinflammation or autoimmunity and thereby modulated the penetrance or expression of traits like attentional volatility or sensory hypersensitivity, rather than deterministically “preventing” them.

By contrast, in post-industrial environments marked by helminth eradication, ultra-processed diets, and widespread antibiotic exposure, this ancient immuno-microbial triad may be destabilized. The result could be a more fragile gut–brain axis, unbuffered by helminthic immunoregulation, contributing—alongside many other factors—to dysbiosis-associated neurodevelopmental conditions such as ADHD or autism spectrum conditions (Bonaz et al., 2018; Reynolds et al., 2015; Bilbo et al., 2018; Hooper et al., 2012). This is not to claim that helminth loss is the primary cause of ADHD, nor that ADHD prevalence has risen solely because of deworming campaigns. Rather, the hypothesis suggests that helminth loss might be one contributor among many to changes in the *expression* and ecological context of neurodivergent phenotypes.

In this model, helminths function as ancient symbiotic sculptors, tuning the gut–brain axis to environmental variability. Their absence in modernity may unmask latent cognitive phenotypes shaped under very different evolutionary rules. Testing this hypothesis would require careful epidemiological and experimental work, including comparisons of ADHD phenotypes in populations with differing parasite exposure histories and mechanistic studies of helminth-induced immune modulation on attention and behavior.

## 4 Helminths as hidden modulators: from invasion to integration

Once established within the host, helminths do not merely evade immune detection; they actively reprogram systemic physiology. Many helminth species secrete immunomodulatory molecules that reshape cytokine profiles, promoting anti-inflammatory mediators such as IL-10 and TGF-β to suppress host responses and ensure their persistence (Maizels and Yazdanbakhsh, 2003; Maizels and McSorley, 2016). These immunological shifts have downstream effects on the central nervous system, partly via enzymes such as indoleamine 2,3-dioxygenase (IDO), which divert tryptophan into the kynurenine pathway.

By accelerating tryptophan degradation into kynurenine and metabolites such as quinolinic acid and kynurenic acid, helminth-induced immune activation can alter serotonergic balance and glutamatergic signaling (Joanna et al., 2017; Savitz, 2020). These alterations have been implicated in a broad spectrum of behavioral outcomes, including irritability, anxiety-like behavior, cognitive slowing, and in some models hyperreactivity. However, the direction and magnitude of these effects depend on multiple factors (metabolite ratios, brain region, developmental timing) and should not be assumed to map linearly onto “hyperactivity” vs. “lethargy”. In the present hypothesis, such shifts are conceived as modulators of network stability and stress responsivity rather than single-cause determinants of ADHD.

Vagal afferents, too, are impacted. Several helminth-derived products can influence afferent vagal tone and autonomic regulation, potentially contributing to lower heart rate variability and altered affective regulation (Bonaz et al., 2018; Foster et al., 2017; McCusker and Kelley, 2013). In children with ADHD, reduced vagal tone and impaired emotional regulation are consistent findings, although they are not specific to ADHD (Beauchaine and Cicchetti, 2019). The possibility that early-life parasitic or microbial immune modulation might entrain long-term autonomic imbalance remains an open and testable question.

In animal studies, helminth infections and related parasitic exposures have been shown to disrupt dopaminergic transmission in the nucleus accumbens and prefrontal cortex—regions critical to reward, attention, and motivation (Williamson and Bilbo, 2013). Rodents infected with *Toxocara canis* or *Trichinella spiralis* exhibit increased anxiety-like behaviors and attention deficits for weeks or months after clearance of the parasite, indicating that even transient colonization can yield persistent neuromodulatory consequences (Guangxu et al., 2018; Quattrocchi et al., 2012). These models are imperfect proxies for human ADHD but illustrate the plausibility of parasite-driven, long-lasting changes in brain function.

From this perspective, parasitic colonization is not a neutral background factor but an active participant in shaping the brain–body interface. The loops of cytokine drift, vagal modulation, tryptophan diversion, and dopaminergic alterations may contribute, in susceptible individuals, to the attentional and emotional volatility characteristic of certain neurodivergent phenotypes.

## 5 Ancestral echoes: epigenetics and transgenerational gut memory

One of the most striking questions raised by the helminth–neurodivergence hypothesis is not merely how parasites affect a given individual, but how their physiological influence might persist across generations, even in the absence of current infection. Epigenetics—heritable modifications of gene expression that do not alter DNA sequences—offers one plausible mechanism (Jaenisch and Bird, 2003; Bohacek and Mansuy, 2015).

Chronic helminthic infections, especially during critical windows of development (prenatal, neonatal, or early childhood), induce systemic immune shifts and gut barrier remodeling. These processes affect the expression of key genes involved in intestinal mucosal architecture, such as MUC2 (coding for mucin-2, a component of the gut's protective mucus layer) and TJP1 (tight junction protein-1, essential for epithelial integrity) (Peterson and Artis, 2014; Rescigno, 2017). Persistent inflammation and altered microbial metabolites can reprogram local and systemic gene expression via DNA methylation and histone modifications (Bohacek and Mansuy, 2015).

Evidence from both murine and human studies suggests that such intestinal and systemic immune perturbations can leave transgenerational marks. In rodent models, early-life inflammation or infection leads to altered methylation at loci governing cytokine production (IL-6, IL-10), serotonergic signaling (SLC6A4, TPH1), and HPA axis regulation (NR3C1), which are then partially transmitted to offspring, who show altered stress responsivity and gut physiology (Hooper et al., 2012; Tamara et al., 2010; Kundakovic and Champagne, 2015; Del Giudice, 2018). In human cohorts, maternal stress and inflammatory states are associated with DNA methylation changes in newborns at genes implicated in stress regulation and mood, including NR3C1, with downstream effects on temperament and vulnerability to psychopathology (Tamara et al., 2010; Kundakovic and Champagne, 2015).

Direct demonstrations that helminth infections in humans produce heritable gut–brain epigenetic signatures are still lacking. Nevertheless, the broader literature on inflammatory and microbial imprinting supports the plausibility of such mechanisms (Gensollen et al., 2016; Hooper et al., 2012). At the same time, these associations are heavily confounded by maternal diet, socioeconomic status, antibiotic exposure, and broader environmental factors, which must be carefully accounted for in future studies.

From this perspective, the “fragile gut” commonly observed in ADHD and related neurodivergent profiles may reflect not only a trait of the individual but a memorial architecture shaped by transgenerational exposure to infection, inflammation, and microbial shifts. The intestinal epithelium and enteric nervous system might retain elements of these altered set-points, shaping immunological tone and neuromodulator availability from the earliest stages of life. This may partially explain the preconfigured susceptibility to both gastrointestinal and cognitive dysregulation seen in certain families, independent of immediate environmental triggers—without implying determinism or excluding psychological and social influences.

In this model, neurodivergence becomes not just a deviation in brain wiring but a systemic legacy, an inherited compromise between immunotolerance and vigilance, forged through millennia of host–parasite cohabitation.

## 6 Evolutionary selection: when fragility was (possibly) functional

Why would a phenotype marked by gut fragility, neuroimmune sensitivity, and attentional instability persist across generations, despite its apparent cost in modern settings? Evolutionary theory invites—but does not compel—consideration of adaptive explanations (Del Giudice, 2018). At the same time, it is critical to distinguish hypothesis-generating narratives from established evolutionary facts.

The persistence of a trait does not, by itself, demonstrate adaptive value. For a trait to be firmly classified as an evolutionary adaptation, one would ideally show (1) heritability, (2) consistent fitness consequences, and (3) genetic signatures of selection. For ADHD, current evidence is incomplete: heritability estimates are moderate to high but variable; fitness advantages are uncertain and likely context-dependent; and genome-wide association studies have not yet yielded clear signatures of positive selection on ADHD risk loci. The present article therefore does *not* claim that ADHD is a proven evolutionary adaptation. Instead, it uses evolutionary reasoning as a conceptual framework to generate testable ideas about gut–brain–immune trade-offs.

Within this heuristic context, one possible scenario is that, in early human ecologies characterized by high pathogen load and environmental unpredictability, certain combinations of intestinal permeability, immune reactivity, and attentional style could have had context-specific benefits. A more porous gut, though vulnerable, may have allowed more rapid antigen sampling and accelerated immune learning in infancy, with gut-associated lymphoid tissue acting as a training ground for tolerogenic and defensive responses (Hooper et al., 2012; Jensen et al., 1997). Likewise, traits such as hypervigilance, rapid attentional shifting, and strong reactivity to novelty—features often seen in ADHD—may have enhanced group-level survival by supplying individuals inclined toward sentinel roles (Hartmann, 1993; Young, 2007; Luo et al., 2022).

Under this model, the gut–brain axis serves as an evolutionary fulcrum: the same intestinal architecture that harbored helminths may have calibrated brains for fast, flexible responses to threat and opportunity. However, these traits likely coexisted with costs (e.g., greater susceptibility to inflammation, sleep problems, or emotional dysregulation), making them candidates for balancing selection or context-dependent neutrality rather than simple “optimizations”.

Crucially, this hypothesis does not romanticize ADHD, nor does it deny the suffering associated with severe forms. Many individuals with ADHD experience significant functional impairment, especially in modern educational and economic systems (Jensen et al., 1997; Luo et al., 2022). An evolutionary framing should be used to highlight *trade-offs and diversity* rather than to minimize disability or to suggest that individuals ought to “accept” under-treatment because their traits were once useful.

## 7 ADHD as a co-evolved profile: a neurointestinal syndrome

Attention Deficit Hyperactivity Disorder (ADHD), often framed as a psychiatric disorder of executive dysfunction or dopaminergic dysregulation, may—in at least a subset of cases—be fruitfully conceptualized as a neurointestinal syndrome: a co-evolved cognitive phenotype shaped by interactions between parasites, microbes, and the immune system. This does *not* imply that all ADHD is gut-driven, nor that brain-centered models are obsolete. Rather, it situates some ADHD presentations within a broader ecological and bodily context.

Multiple studies have demonstrated altered gut microbiota composition in individuals with ADHD, with reduced alpha-diversity, lower abundance of SCFA-producing bacteria, and imbalanced *Clostridium* and *Bacteroides* species (Aarts et al., 2017; Prehn-Kristensen et al., 2018; Alverina et al., 2021; Dalile et al., 2019). At the same time, other studies report smaller or inconsistent differences, underscoring the heterogeneity of findings and the likelihood that only a subset of ADHD is strongly microbiome-associated (Martami et al., 2018; Alverina et al., 2021; Bastiaanssen and Cryan, 2021). Reviews of the field emphasize modest effect sizes and the need for larger, better-controlled cohorts (Alverina et al., 2021; Bastiaanssen and Cryan, 2021).

These microbial profiles, when present, correlate with increased intestinal permeability, a pro-inflammatory milieu, and disrupted tryptophan–serotonin–kynurenine metabolism, all of which are capable of influencing dopaminergic signaling and emotional regulation (Fasano, 2012; Magistris et al., 2010; Dalile et al., 2019; Joanna et al., 2017). Importantly, such traits often emerge early in life, in parallel with neurodevelopmental trajectories, suggesting they may be part of the same developmental ecology rather than simple comorbidities. Whether the gut component shares the high heritability observed for core ADHD symptoms remains unclear.

Simultaneously, helminths, ubiquitous in ancestral environments, modulated this gut–brain system in ways that might have favored neuroplasticity and flexible cognition in some contexts. Their presence induced immunological tolerance via IL-10 and TGF-β, shaped vagal tone, and altered the brain's arousal circuitry (Bonaz et al., 2018; Maizels, 2020; Maizels and Yazdanbakhsh, 2003; Maizels and McSorley, 2016). Such immune education could, in theory, have supported cognitive profiles characterized by rapid reactivity, risk-taking, environmental scanning, and novelty-seeking—traits today associated with ADHD (Hartmann, 1993; Young, 2007; Luo et al., 2022). Again, this remains a hypothesis, not a demonstrated evolutionary path.

In this evolutionary context, ADHD-like behaviors may have been adaptive in pathogen-rich, unstable ecologies for some individuals and some dimensions of the phenotype, while being neutral or disadvantageous in others. In contemporary post-industrial societies, where helminths have been eliminated and microbial diversity is diminished due to antibiotics, hygiene, and urban living, this ancient co-adaptation may be mismatched. The “sentinel brain” now inhabits sedentary, overstimulating classrooms and cognitively homogenizing institutions, rendering its traits disruptive rather than adaptive.

It is equally important to recognize that many individuals with ADHD have no obvious gastrointestinal symptoms, no documented gut abnormalities, and respond robustly to standard stimulant medications targeting brain dopamine. This does not contradict the present hypothesis; rather, it highlights that ADHD is highly heterogeneous, encompassing multiple subtypes and pathways. The neurointestinal model is best viewed as one mechanistic lens among others, particularly relevant for individuals with prominent gut symptoms, inflammatory markers, or microbiome alterations.

Therapeutically, this framework does not suggest replacing existing evidence-based treatments. Stimulant medications and psychosocial interventions remain first-line and often highly effective. Instead, it motivates research into adjunctive strategies that target the gut–brain axis—such as probiotics, prebiotics, dietary interventions, lifestyle changes, and potentially vagus nerve modulation—in carefully phenotyped subgroups of ADHD. Work on gut microbiome–based interventions in inflammatory bowel disease and other immune-mediated conditions illustrates how restoring microbial resilience and barrier integrity can have systemic benefits (Hetta et al., 2024). Whether similar strategies can meaningfully modulate ADHD symptoms is an open, testable question that will require rigorous randomized trials.

Finally, physiopathological study of secondary intestinal reinfestation of a deparasitized digestive tract (particularly in Western countries) by intestinal worms acquired during foreign travel, and its repercussions on the intestine–brain axis and ADHD symptomatology, could provide a natural experiment to probe aspects of this hypothesis—again, with careful attention to safety, ethics, and confounding factors.

Thus, for at least a subset of individuals, ADHD may be less the result of isolated brain dysfunction and more a broader *mismatch syndrome*, a gut–brain dissonance between evolved ecological intelligence and modern neurocultural expectations.

## 8 Conclusion: from comorbidity to codependence—and to testable hypotheses

The neurodivergent gut is not an incidental vulnerability; it is a structural participant in the orchestration of cognition. Its fragility, when present, may reflect an evolutionary trade-off: greater openness and responsivity in exchange for increased susceptibility to dysregulation. In this Perspective, I have proposed that ADHD in a subset of individuals can be framed as a neurointestinal phenotype emerging from long-term interactions between microbes, helminths, and host immunity, and now rendered partly maladaptive by rapid cultural and ecological change.

To avoid conflating hypothesis with fact, it is essential to translate this narrative into testable predictions:

1. Phenotypic stratification
2. ADHD populations could be stratified based on gut barrier markers (e.g., zonulin), microbiome composition, and inflammatory profiles. The hypothesis predicts that individuals with pronounced intestinal permeability and dysbiosis will show distinct cognitive and affective patterns and may respond differently to gut-targeted interventions than those without such features.
3. Ecological and historical comparisons
4. Cross-cultural and epidemiological studies could compare ADHD prevalence, symptom structure, and gut phenotypes in populations with differing histories of helminth exposure, deworming campaigns, diet, and antibiotic use. Rather than assuming a simple rise in ADHD due to helminth loss, this approach would seek subtle shifts in comorbidity patterns, severity, or treatment response.
5. Mechanistic and interventional studies
6. Experimental models and clinical trials could assess whether specific microbiome-based interventions (e.g., targeted probiotics, prebiotics, dietary modulation, fecal microbiota transplantation in extreme cases) preferentially benefit ADHD subgroups defined by gut abnormalities—and whether such effects are mediated by changes in SCFAs, kynurenine pathway metabolites, or vagal tone.

Clinically, the framework encourages an integrative, ecological psychiatry that complements—not replaces—established neurobiological models. It underscores the need for humility: reinterpretations of ADHD as “adaptive” must not obscure the genuine impairment many individuals experience, particularly those with severe presentations, comorbidities, or socioeconomic disadvantage.

On a deeper level, the hypothesis invites us to consider that aspects of human attention, impulsivity, and emotionality may not be fully understandable without the gut. The intestinal wall is more than a barrier; it is a historical interface where microbes, parasites, and host tissues negotiated the terms of coexistence—and, perhaps, inscribed some of the variability we now call neurodivergence.

## Data Availability

The original contributions presented in the study are included in the article/supplementary material, further inquiries can be directed to the corresponding author.
